# A new detector concept based on the prompt gamma radiation analysis for *In vivo* boron monitoring in BNCT

**DOI:** 10.1093/rpd/ncac245

**Published:** 2023-10-11

**Authors:** M Silarski, K Dziedzic-Kocurek, F Sobczuk, A Nykiel, P Moskal, S Niedźwiecki, E Ł Stępień, M Szczepanek

**Affiliations:** Faculty of Physics, Astronomy and Applied Computer Science, Jagiellonian University, PL-30-348 Krakow, Poland; Faculty of Physics, Astronomy and Applied Computer Science, Jagiellonian University, PL-30-348 Krakow, Poland; Faculty of Physics, Astronomy and Applied Computer Science, Jagiellonian University, PL-30-348 Krakow, Poland; Institute of Nuclear Physics, Polish Academy of Sciences, PL-31-342 Krakow, Poland; Faculty of Physics, Astronomy and Applied Computer Science, Jagiellonian University, PL-30-348 Krakow, Poland; Center for Theranostics, Jagiellonian University, PL-31-501 Krakow, Poland; Faculty of Physics, Astronomy and Applied Computer Science, Jagiellonian University, PL-30-348 Krakow, Poland; Faculty of Physics, Astronomy and Applied Computer Science, Jagiellonian University, PL-30-348 Krakow, Poland; Center for Theranostics, Jagiellonian University, PL-31-501 Krakow, Poland; Faculty of Physics, Astronomy and Applied Computer Science, Jagiellonian University, PL-30-348 Krakow, Poland

## Abstract

The problem of boron concentration monitoring during the boron neutron capture therapy (BNCT) therapy is one of the main challenges of this type of radiotherapy and is directly related to the nature of the interaction of neutrons with mater. Among the available *in vivo* methods of boron monitoring positron emission tomography seems to be very promising but it requires a new boron carrier with a β^+^ emitter, which is not yet clinically available. An alternative solution may be the prompt gamma radiation analysis (PGRA) based on the secondary radiation emitted in the interaction of neutrons with the patient’s tissues. This method requires, however, compact gamma radiation detection systems sustaining high counting rates and characterized by very good energy resolution. In this contribution, we present state-of-the-art solutions for monitoring in BNCT based on PGRA. Moreover, we describe a new concept of such a system based on position-sensitive scintillator detectors equipped with an anti-Compton shield and data analysis supported with modern artificial intelligence algorithms.

## Introduction

The boron neutron capture therapy (BNCT) is a radiation-based treatment modality used mainly against melanoma, as well as head, neck and brain cancers, especially glioblastoma multiforme. The latter is one of the most challenging malignancies to treat for which BNCT appears to be much more effective than the other therapies^(1)^. Its main advantage is the selectivity of tumor cells destruction by ‘labeling’ them with the ^10^B isotope characterized by one of the highest cross-sections for thermal neutron capture^(2)^. This reaction results in the creation of an α particle and ^7^Li nucleus, which deposit their energy (∼3 MeV) within a distance comparable to size of a single cell. At the same time, the healthy cells which do not contain boron are exposed to a much smaller radiation dose. For a long time, the use of BNCT was limited due to the lack of intense epithermal neutron fluxes available at nuclear reactors only. This problem has recently been solved with sources based on proton accelerators producing high-intensity neutron fluxes via the ^7^Li(p,n)^7^Be or ^9^Be(p,n)^9^B nuclear reactions^(3)^, which can be installed at hospitals. There are two major issues in BNCT, which still should be addressed. One is delivering boron to the tumor cells with a subsequent lowest possible concentration in the healthy tissues. Thus, a lot of effort is devoted to research new, more effective ^10^B carriers^(4, 5)^. The other problem in BNCT therapy is the monitoring of boron concentration in the patient’s body, especially during the therapeutic irradiation, and delivered dose estimation. So far the only *in vivo* boron distribution measurements have been done using positron emission tomography (PET), which requires the boron carrier to be modified and labeled with a β^+^ emitter. Hitherto, the PET examination is done to determine the boron distribution before the irradiation to verify the treatment plan. The application of this imagining modality is limited in use for real-time monitoring due to the high background originating mainly from the interaction of the 2.2-MeV gamma quanta from neutron capture on hydrogen, which interact with the patient’s body. The other problems are the limited field of view of presently used PET scanners and the much higher activity of the β^+^ tracer administered together with the boron carrier to the patient^(4)^. Here, the solution may be provided by total-body PET scanners able to image the whole body of a patient with better sensitivity and resolution^(6–8)^. The other solution for the boron concentration real-time monitoring may be the prompt gamma radiation analysis (PGRA). It is based on a registration of γ quanta emitted as a result of the therapeutic neutron beam interactions with the tumor and surrounding tissues. In practice, there are two γ quanta that could be used: the 478 keV originating from the neutron capture on ^10^B and the 2.2 MeV produced in the same reaction on ^1^H. Thus, the 478-keV gamma rays may be used to determine the boron distribution using e.g. the single-photon emission computed tomography (SPECT) or Compton camera solutions. Some of the already developed PGRA-based systems are presented in the next section together with issues that still need to be addressed before they can be used in the clinical routine.

## Prompt gamma radiation analysis in bnct

The BNCT requires intense neutron flux in the epithermal energy range of 0.5 eV–40 keV^(2)^. These neutrons thermalize while reaching the tumor and healthy tissues in its vicinity by multiple elastic scattering and may be captured. Some of these reactions lead to the emission of secondary γ quanta with energies characteristic to the isotope, which captured the neutron. Among all the basic constituents of tissues, hydrogen has the highest cross-section for the radiative neutron capture, which is of about three orders of magnitude higher than e.g. the value for carbon^(9)^. The ^10^B(n,α)7Li nuclear reaction, being the basic therapeutic process in BNCT, is also accompanied by γ radiation emission (in ~94% of the cases^(4)^). Thus, during the therapy one should expect the emission of secondary γ radiation with two energies: 2.2 MeV (hydrogen) and 478 keV (boron). Both of them may be used to estimate the distribution of the boron therapeutic dose and the dose due to γ radiation. At the same time, registration of the boron line may give information about the concentration of the ^10^B isotope itself. In practice, the reconstruction of emission positions of the 478-keV γ quanta is quite a challenge. One of the reasons is a high background originating from the pair creation occurring in the body for the 2.2-MeV hydrogen line. It generates secondary gamma rays with energy equal to 511 keV, which may overlap with the boron signal. This puts stringent conditions on the detectors to be used in the monitoring systems, which should be characterized by energy resolution high enough to separate these two energies. The other major requirement is high detection efficiency since boron content in BNCT is at the level of tens of microgram per gram of tumor and the expected signal statistics is low. There are two imagining modalities used so far in the PGRA for BNCT: Compton cameras and single photon emission computed tomography (SPECT) on which we focus in this article. The requirements of high-efficiency and sufficient energy resolution for registration of the 478-keV gamma rays limit the possible detection materials and geometry for a successful on-line monitoring system for BNCT. The natural choice would be the High purity Germanium (HpGe) detectors offering the best energy resolution (<1%)^(10)^. They may be produced in relatively large dimensions and their density is high (>5 g/cm^3^). These detectors exhibit, however, several drawbacks and so far they are not used for BNCT monitoring^(11)^. First of all, they need cooling (usually with liquid nitrogen in a cryostat), which decreases their mobility and limits geometric arrangement. Moreover, HpGe detectors are sensitive to neutron fields, which may decrease their performance or even damage the crystals. The other detection materials, which may be considered are the CdTe and CdZnTe (CZT) semiconductors with only slightly worse energy resolution^(10)^. Moreover, they offer possibility to construct much more compact detection systems operating with a small Peltier cooler^(11)^. Several attempts have been made to develop the BNCT monitoring system based on the CZT detectors^(11–13)^. All of them have similar issues connected mainly to the small sizes of crystals available commercially for which thickness do not exceed few centimeters^(13)^. This reduces the efficiency of the detection system and increases the number of electronic channels to be read out in case of bigger monitoring systems. Moreover, the detector material itself may be a source of background due to radiative neutron capture on cadmium, which additionally decreases the signal-to-noise ratio^(13)^. Alternative solutions are based on scintillating crystals offering much higher detection efficiency, but at the same time in most cases much worst energy resolution^(10)^. Several PGRA-SPECT systems based on scintillators were proposed and tested. Kobayashi *et al*. suggested that with a proper collimator design it is possible to use even BGO crystals with one of the shortest mean free paths for 478-keV photons and not so good energy resolution (∼14%^(11)^). Other proposed solutions are based on LaCl_3_:Ce^(14)^ and CsI(Tl) crystals^(15)^ having a much smaller density than the previously mentioned scintillators, which may be compensated by larger dimensions. Unfortunately, so far none of the mentioned designs provides the spatial resolution and signal-to-noise ratio acceptable during the clinical irradiation.

## New detector concept based on lanthanum bromide

Another promising scintillator that may be used to construct a monitoring system for BNCT is lanthanum bromide (LaBr3:Ce) and its enhanced version LaBr_3_:Ce:Sr^(16)^. It is the brightest scintillator characterized by the best energy resolution (∼4% for the 511-keV γ quanta) and efficiency comparable to the one of HpGe detectors^(17)^. Thus, it is often considered as a cheaper and easier-to-operate alternative to semiconductors in many applications, e.g. homeland security^(18)^. Drawbacks of this detection material are its hygroscopicity and internal radiation, which may affect detection of low activity samples in the energy range up to almost 4 MeV, especially in the case of large detectors, e.g. 3″ × 3″.

**Figure 1:**
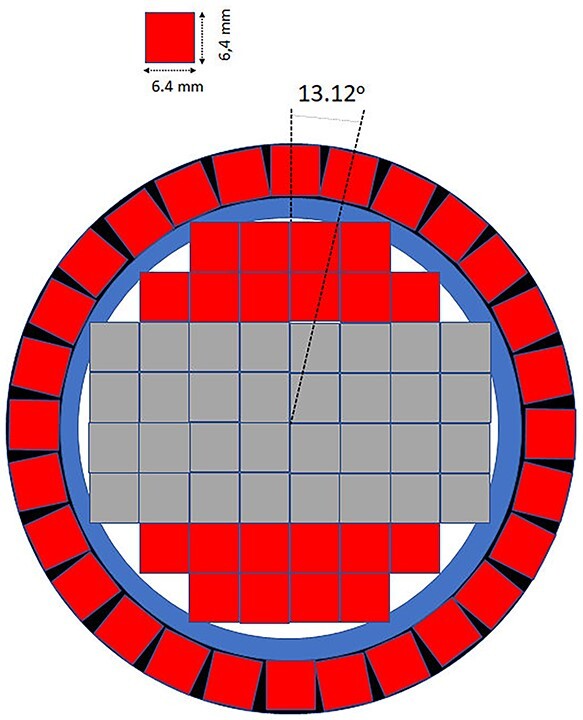
Schematic view of the cross-section of the first prototype of a detector for BNCT monitoring system based on the LaBr_3_:Ce:Sr scintillator with the position-sensitive light readout. Red squares show single silicon photomultipliers, whereas the gray ones represent their matrices. Blue and black colors show the mechanical support for the anti-Compton shield and the crystal casing, respectively.

**Figure 2:**
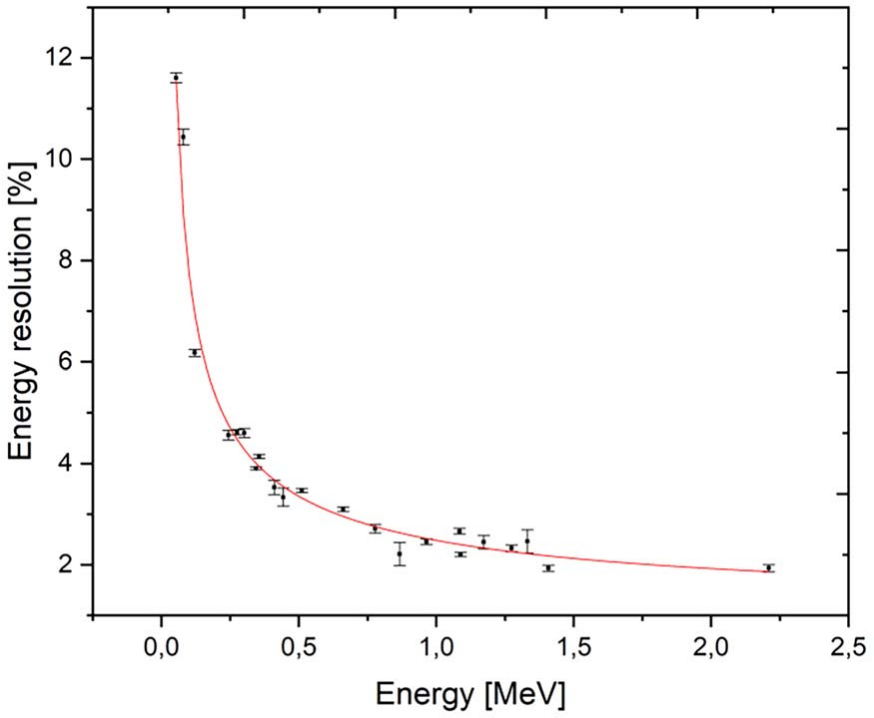
Preliminary results of the LaBr3:Ce:Sr scintillator energy resolution studies in the energy range up to 2.2 MeV. The highest energy line was generated by AmBe source neutrons captured on hydrogen. The red line represents fit to the data using the R = (a + bE + cE^2^)^1/2^ function. Figure adapted from^(20)^.

For the construction of the first PGRA-SPECT prototype based on the LaBr3:Ce:Sr scintillator we decided to use a 2″ × 2″ crystal with the silicon photomultipliers light readout and anti-Compton shielding. In the first prototype, the readout system have been constructed with Hamamatsu S14160-6050HS silicon photomultipliers with dimensions of 6 × 6 mm^2^ covering the optically opened crystal side as shown schematically in Figure 1. The photosensors in the central part of the crystal are gathered in two 4 × 4 matrices to maximize the active part sensing the scintillating light. The application of silicon photomultipliers allows for the construction of much more compact monitoring system and for estimation of a position of *γ* quantum reconstruction in the readout plane. To suppress background originating from the Compton interactions of gamma rays in the detector and to increase the signal-to-background ratio we decided to construct an anti-Compton shielding made from 28 BGO crystals with dimensions 6 × 6 × 60 mm^3^, which surround the side of the main crystal. This idea was already used with success in the development of noninvasive sensors of illicit materials^(19)^ and in one of the monitoring systems based on CdTe detectors^(13)^. This detector will be working in an anti-coincidence mode with the signals from the main crystal, which allows rejecting Compton scattered gamma rays that interacted also in the BGO shield with efficiency of at least 75% at the energy of 478 keV. It may also reduce background due to radiation hitting the detector from the side. The detector is still under construction and in parallel we have been performing research on the characterization of the LaBr3:Ce:Sr crystal we intend to use in the monitoring system read out by a Hamamatsu R7723-100 photomultiplier. We have studied the energy and time resolutions, light output and the linearity of the detector response^(20)^. The energy resolution was measured using isotopic radiation sources up to about 1.5 MeV and a neutron induced 2.2-MeV hydrogen line (see Figure 2). We have confirmed, that the enhanced lanthanum bromide crystal offers the best energy resolution among all the available scintillators for energies close to the 478 keV (~3.3%) and for hydrogen γ quanta (∼2%). The time resolution of the detector was studied using a collimated ^22^Na source and BaF_2_ reference detector giving the value of 470 ± 5 ps^(20)^.

## Summary and outlook

One of the major issues in the BNCT is the lack of real-time monitoring of delivered dose and boron concentration. Despite several trials, there is no such a device that would fulfill this task in a clinical use. The main problem in effective BNCT monitoring is a high background originating mainly from the thermal neutron capture on hydrogen. This is one of the sources of difficulties in applying the PET imagining during the therapy. The other imagining modalities which may be used, PGRA-SPECT and Compton cameras, require high-efficiency detectors providing separation of the 478 and 511-keV lines. An interesting alternative for detectors based on CdTe and CdZnTe semiconductors may be inorganic scintillator LaBr_3_:Ce:Sr providing very good energy resolution and efficiency comparable to the HpGe detectors. Thus, we have proposed to use monolithic lanthanum bromide crystals with a silicon photomultipliers light readout with an anti-Compton shield. In the first prototype we used 2″ × 2″ crystal read out by 62 SiMPs, which may enable the determination of a place of γ quantum interaction with a few millimeters of precision using neural network algorithms^(21)^. This possibility, together with the rejection of Compton scattered γ quanta background may significantly decrease the background and increase the signal-to-background ratio for our PGRA-SPECT system being under construction. In parallel, we have performed characterization of the used LaBr_3_:Ce:Sr crystal confirming its superior energy resolution for both, the boron and hydrogen lines, and very good timing properties.
